# Exploring readiness for advance care planning in Japan: A qualitative interview study with older adults with frailty, family caregivers, and health and social care practitioners in the homecare setting

**DOI:** 10.1177/26323524251395654

**Published:** 2025-11-28

**Authors:** Miki Fujimoto, Jonathan Koffman, Ito Nagata, Yukihiro Sakaguchi, Catherine J. Evans

**Affiliations:** 1 Cicely Saunders Institute of Palliative Care, Policy and Rehabilitation, Florence Nightingale Faculty of Nursing Midwifery and Palliative Care, King’s College London, UK; 2 Wolfson Palliative Care Research Centre, Hull York Medical School, UK; 3 College of Nursing Art and Science, University of Hyogo, Kobe, Japan; 4 School of Human Welfare Studies, Kwansei Gakuin University, Nishinomiya, Japan; 5 Sussex Community NHS Foundation Trust, Brighton, UK

**Keywords:** advance care planning, aged, frail elderly, homecare services, qualitative research

## Abstract

**Background:**

Older adults with frailty are at risk of rapid health decline and poor outcomes, including end of life. While advance care planning (ACP) can support person-centred care, how to assess and support older adults’ ACP readiness remains unclear. Health and social care practitioners, and family caregivers, are often main providers of care, but their role in ACP is poorly understood.

**Objectives:**

To understand the readiness of older adults with frailty to engage in ACP from the perspectives of older adults, family caregivers, and health and social care practitioners.

**Design:**

An exploratory qualitative interview study informed by the COM-B system (individual-level behaviour change) and the Ecological Systems Theory (system-level influences).

**Methods:**

Semi-structured qualitative interviews with older adults (mild to moderate frailty), unpaid family caregivers, and health and social care practitioners in Japan. The topic guide was informed by the theoretical frameworks, exploring individual- and system-level factors influencing readiness. Reflexive thematic analysis was conducted to generate themes within and across the participant data sets.

**Results:**

Twenty-nine interviews were conducted with 32 participants: older adults (*n* = 10), caregivers (*n* = 6), and health (*n* = 6) and social care practitioners (*n* = 10). Three themes were generated: (1) ‘*Leave decision-making to family*’ and Bridging family dynamics – older adults delegate decisions, practitioners navigate complex family relationships; (2) ‘*ACP is not part of our rol*e’: Diverging role expectations among practitioners, and hesitation to engage in ACP – differing perceived practitioners responsibilities limit willingness to initiate ACP; (3) Transitional period: Social norms around death and dying, family structures, and health and social care systems – shifts in systemic and social aspects shape ACP readiness.

**Discussion:**

ACP readiness among this population is not solely an individual decision but is shaped by relationships, systemic, and societal factors. Enhancing ACP readiness requires a system-wide approach that incorporates family involvement and interdisciplinary collaboration and is adapted to the local context.

**Trial registration:**

Not applicable.

## Background

Frailty in older adults is characterised by reduced physiological reserves, leading to an increased vulnerability to decline from minor stressors and a higher risk of poor outcomes, including loss of function and end of life.^1–3^ Multiple conditions are also common,^4,5^ becoming more prevalent with age and closely linked to frailty.^6^ An uncertain illness trajectory is inherent among adults with frailty, making prognosis challenging.^7^ Given this uncertainty, they often have unmet palliative care needs.^8,9^

The East Asia and Pacific region is rapidly ageing, with several countries already ‘super-aged’ society (over 20% of the population aged 65 or older) or nearing this status.^10,11^ Japan, the most super-aged society, has 29.1% of its population aged ⩾65 years in 2024.^12^ The advancement of population ageing is likely contributing to an increase in the number of older adults living with frailty – a trend that is projected to continue globally.^13^

Given their uncertain trajectories and risks, older adults with frailty may particularly benefit from advance care planning (ACP).^14^ ACP offers ongoing opportunities for individuals to discuss and plan their current and future care with trusted family members and healthcare practitioners.^15,16^ It is also one of the core components of palliative care,^8^ and can help address the unmet palliative care needs commonly experienced by this population. However, ACP remains poorly understood among this population,^14,17^ and most existing studies have focused on disease-specific populations, such as those with cancer or dementia.^18–21^

To enable meaningful engagement with ACP, it is important to first consider individuals’ readiness to participate in ACP. Readiness for ACP is defined by two elements: (1) *willingness* – the desire to engage in ACP and (2) *ability* – the capacity to do so.^22,23^ Readiness is essential for meaningful engagement,^16^ as a lack of readiness can lead to harmful and confusing conversations.^24^ Understanding of ACP readiness, including how it is experienced, perceived, facilitated, and assessed in routine care, remains limited.^25,26^

Although each context has different names and roles, social care practitioners, such as homecare managers, social workers, and care workers generally support clients through life transitions,^27,28^ even before the onset of illness. They are typically more familiar with their clients’ preferences than, for example, healthcare practitioners.^27^ Owing to their close and continuous contact with clients, social care practitioners may be well positioned to recognise signs of deterioration^28^ and initiate conversations about future care.

In Japan, homecare managers are social care practitioners, the majority of whom (75.6%) are care workers.^29^ They play a central role in the Long-Term Care Insurance System^30,31^ and are responsible for planning and coordinating care and services.^30^ Through regular home visits, they often develop close relationships with older adults. Under the Long-Term Care Insurance System, interdisciplinary team work, such as health and social care practitioners through the Community-based Integrated Care System,^32^ which is comprehensive community-based support and service delivery system to community-dwelling older adults. Moreover, the Guideline for the Decision-Making Process of the End-of-Life Medical care was revised in 2018 to emphasise the importance of interdisciplinary teamwork, including homecare managers, in supporting decision-making processes.^33^

However, ACP remains underemphasised within their professional role as homecare managers.^34^ Little is also known about their integrated work with healthcare practitioners to support ACP.^34^ This includes key community-based healthcare practitioners, notably home-visit nurses, who deliver medical and daily living care at home, and homecare physicians, who make regular and unscheduled visits to patients receiving home-based care.^32,35^

This study aims to understand the readiness of older adults with frailty to engage in ACP from the perspectives of older adults, family caregivers, homecare workers, home-visit nurses, and homecare physicians. The objectives are (1) to understand the experiences and perspectives of older adults, family caregivers and healthcare and social care practitioners on readiness for ACP and (2) to explore the facilitators and barriers to readiness for ACP. The study is conceptually informed by the COM-B system^36^ and the Ecological Systems Theory,^37^ which together provide a theoretical lens for understanding how individual behaviours and wider systemic factors may shape ACP readiness.

## Methods

### Study design and theoretical underpinning

This exploratory qualitative interview study investigates older adults’ readiness for ACP. Readiness is defined as the willingness and ability to take action^22,23^ and is understood as part of the behaviour change process.^38,39^ The study is informed by two theoretical frameworks (Table 1 outlines both frameworks and their respective applications). The first is the COM-B system,^36^ which explores individual-level factors, including capability, opportunity, and motivation. However, an individual behaviour change perspective alone does not fully capture older adults’ readiness for ACP, as ACP is influenced by external factors and follows a dynamic process.^40^ To address this, the Ecological Systems Theory is also used to consider system-level factors.^37^ Reporting follows the Reflexive Thematic Analysis Reporting Guideline (RTARG) (Supplemental Appendix 1).^41^ RTARG was specifically developed to guide and evaluate studies using reflexive thematic analysis, as existing checklists such as the Consolidated Criteria for Reporting Qualitative Studies (COREQ)^42^ are not epistemologically aligned with this approach, being underpinned by (post) positivist assumptions.^41^

**Table 1.** Underpinning theories: COM-B system and ecological model.

Underpinning theories	Description
COM-B system^36^	This model identifies three interacting conditions that determine behaviour: ***Capability*** – an individual’s psychological and physical capacity to engage in the activity; ***Opportunity*** – external factors that enable or prompt the behaviour, including physical, social, and environmental influences; ***Motivation*** – the internal processes that direct and energise behaviour, including habitual responses, emotional reactions, and conscious decision-making. These conditions interact dynamically: *capability* and *opportunity* influence *motivation*, and performing a behaviour can, in turn, affect all three conditions. Behaviour change may be triggered by changes in any one condition, though all three may be required to achieve and sustain a specific behavioural goal.
Ecological Systems Theory^37^	It offers a comprehensive framework for understanding how individual behaviour is shaped by multiple, interacting layers of influence within their environment. It comprises five interrelated levels: ***Microsystem*** – the immediate environment where the developing person engages in face-to-face interactions, shaped by activities, social roles, and interpersonal relationships, with specific physical, social, and symbolic features that encourage or restrict engagement; ***Mesosystem** –* the connections and interactions between two or more settings that directly involve the developing person; ***Exosystem*** – the linkages and interactions between two or more settings, where at least one does not directly include the developing person, but events in that setting indirectly affect their immediate environment; ***Macrosystem*** – the overarching patterns of cultural, societal, and systemic influences, encompassing belief systems, resources, customs, lifestyles, and societal structures; ***Chronosystem*** – changes or consistencies over time in both the characteristics of the person and the environment in which they live.

### Public involvement and stakeholder consultation

Two older adults with frailty (one male and one female) were involved as lived-experience experts. Additionally, two homecare managers and a home-visit nurse working at the research site were invited to a stakeholder consultation. Specifically, we sought their advice on the study procedures and all related documents.

### Setting and participants

This study was conducted in a city in Japan with a population of 99,037, of whom 26.5% are aged 65 years or older in 2025,^43^ classifying it as a super-aged society.^11^ The participants are older adults with frailty residing at home, their unpaid family caregivers, social care practitioners (homecare managers), and healthcare practitioners (home-visit nurses and homecare physicians; Table 2 details the eligibility criteria). The level of frailty was defined as living with pre-frailty or greater, based on the Japanese-adapted Japan Frailty Scale, with a score of 4 or above.^44^ These multiple stakeholders were included to gain an in-depth understanding of older adults’ readiness for ACP from the perspectives of all those involved.^45,46^

**Table 2.** Inclusion and exclusion criteria of participants.

Criteria	Older adults with frailty	Family caregivers	Professionals
Inclusion	• Aged 65 and over • Living at home in the study site • Identified as living with mild frailty and above based on the Japanese-adapted Japan Frailty Scale (score of 3 and above)^44,45^ • Decisional capacity to consent and undertake an individual interview assessed using Mental Capacity Act criteria^47 *^	• Aged 18 and over • Currently caring for or had cared for older adult with frailty • Decisional capacity to consent and participate in an individual interview was assessed using the Mental Capacity Act criteria^47 *^	• All types of health and social care professionals: homecare doctors, home-visiting nurses, and care managers • Providing primary healthcare, home and/or community care services for older adults with frailty
Exclusion	• Too unwell to participate in an interview, as advised by the care manager • Hospitalised or living in a nursing home	• Too unwell to participate in an interview, as advised by the care manager or older adult	

* Potential participants with dementia or cognitive impairment were not excluded, in alignment with the principles of the Mental Capacity Act.^46^ A process consent approach was applied to maximise individual autonomy with support from consultees such as health and social care practitioners and family caregivers.

### Selection of participants

Purposive sampling was employed across all participant groups to maximise variation in the participants, based on characteristics including gender, level of frailty (mild or above), living arrangement (e.g. living alone), family involvement, and year of experience. Information power was used to determine the number of participants.^48^ It suggests that the sufficiency of a sample depends on the aim of the study, sample specificity, use of established theory, quality of dialogue, and analysis strategy. The concept aligns well with reflexive thematic analysis, which was applied in this study^49^ and has been used in similar qualitative studies.^50–53^ The planned sample comprised 10 older adults, 8 family caregivers, 10 homecare managers, 3 home-visit nurses, and 3 homecare physicians.

### Recruitment strategies

Homecare workers and home-visit nurses identified potentially eligible older adults and family caregivers using study criteria and approached them with a flyer. Older adults were asked if their family caregivers might also participate. Potential participants either contacted the researcher (M.F.) directly or gave verbal consent for practitioners to share their contact details with M.F. Flyers were also displayed in communal spaces, such as local community centres, to encourage direct contact. M.F. assessed participants’ capacity to consent using the Mental Capacity Act criteria^47^ and obtained written informed consent.

Homecare workers, home-visit nurses, homecare physicians were recruited through the Comprehensive General Support Center in the city,^31^ a community-based institution that provides integrated support services for older adults. They either contacted M.F. directly or consented to the organisation to share their contact details. M.F. then obtained written consent.

### Data collection

Semi-structured, in-depth interviews were conducted to explore experiences and perspectives on readiness for ACP among older adults, either individually or in dyads (older adult/family caregiver), guided by an interview topic guide (Supplemental Appendix 2). Development of the guide was informed by the framework for devising a qualitative semi-structured interview guide,^54^ drawing on existing literature on readiness for ACP among community-dwelling older adults with frailty, and informed by two theoretical frameworks: the COM-B system of behaviour change^36^ and an Ecological Systems Theory.^37^ The guide encompassed key aspects of readiness, including the willingness and ability to engage in ACP, as well as perceived facilitators and barriers at multiple levels from individual to systemic. To ensure content validity and cultural appropriateness, the topic guide was reviewed through public involvement and stakeholder consultation.

Interviews took place at participants’ homes, in a private meeting room in a public organisation, or online for some practitioners, ensuring a safe and comfortable setting. M.F., a Japanese female registered nurse with qualitative research experience, conducted all interviews in Japanese, which were digitally recorded and transcribed verbatim. Her prior clinical experience and assumptions about the benefits of ACP and the importance of involving multiple stakeholders shaped the initial focus of the study. These influences were acknowledged and critically reflected upon throughout the research process, particularly through regular discussions with the research team. M.F. also maintained field notes and reflexive journal,^55^ supported by C.J.E. and J.K., both of whom have extensive experience in qualitative research.

### Data analysis

Data analysis employed reflexive thematic analysis,^55^ suitable for capturing the depth and complexity of subjective experiences of readiness for ACP. Its flexibility enables researchers to engage deeply with participants’ narratives, allowing for an understanding of the nuanced and dynamic factors influencing readiness for ACP. Data management and analysis were conducted in Japanese using NVivo software (version 14).

Analysis began with M.F. inductively coding data related to readiness for ACP, iteratively refining codes to ensure they reflected the subtle perspectives of the different participant groups. For dyadic interviews, both participants’ responses were transcribed as a single data source, but their individual contributions were identified and coded separately where distinguishable. Joint or co-constructed responses were also noted to capture relational meaning-making. This dual attention to individual and shared perspectives enabled a more nuanced understanding of how family dynamics and communication influenced readiness for ACP.

In the next stage, related codes were clustered to form sub-themes, each representing a distinct yet connected dimension of the overarching concept. The identification of sub-themes involved examining patterns of meaning within the coded data and comparing them across cases regarding readiness for ACP. Sub-themes were iteratively refined to ensure they were analytically distinct, internally coherent, and contributed meaningfully to the developing interpretation. Sub-themes were then synthesised into themes that captured shared meaning and were organised around central concepts, moving beyond topic summaries towards interpretative depth. This process was also undertaken iteratively, with themes being refined to ensure analytical coherence and conceptual clarity.^55^

Crystallisation, a form of expanded triangulation,^41,46,56^ was employed to systematically explore both convergence and divergence in participants’ views across participant groups. This involved generating both semantic and latent codes^55^ and actively comparing accounts within and between groups to highlight how readiness for ACP was similarly or differently constructed. Subsequently, two theoretical frameworks^36,37^ were applied deductively to structure and deepen thematic development. Themes were carefully generated and named to ensure they encapsulated shared meaning, were united around a central organising concept, and reflected a deeper level of interpretation, going beyond mere topic summaries. Themes and codes were repeatedly reviewed and refined by M.F. to ensure accuracy and coherence.

The process of data analysis was supported by a home-visit nurse (I.N.), who contributed to both coding and interpretation, drawing on over a decade of experience caring for older adults with frailty in the research setting. A psychology researcher (Y.S.) provided feedback on data interpretation, particularly about behaviour change perspectives. C.J.E. and J.K., experts in reflexive thematic analysis, provided critical feedback on the analytic process. Themes, codes, and illustrative quotations were translated into English by M.F. and reviewed collaboratively with J.K. and C.J.E. Any differences in interpretation or coding were resolved through reflexive discussions. The translated themes and quotations were back-translated into Japanese by a researcher (K.M.)^57^ to ensure linguistic consistency and conceptual credibility. This process supported the credibility of the findings by confirming that the meanings remained faithful to the participants’ original expressions.

### Ethical approval

This study received ethical approval from the Research Ethics Committee, King’s College London (HR/DP-22/23-34030) and the Committee for Regulations for Behavioural Research with Human Participants in Kwansei Gakuin University (2022-90).

## Results

### Participants

**Figure 1.**
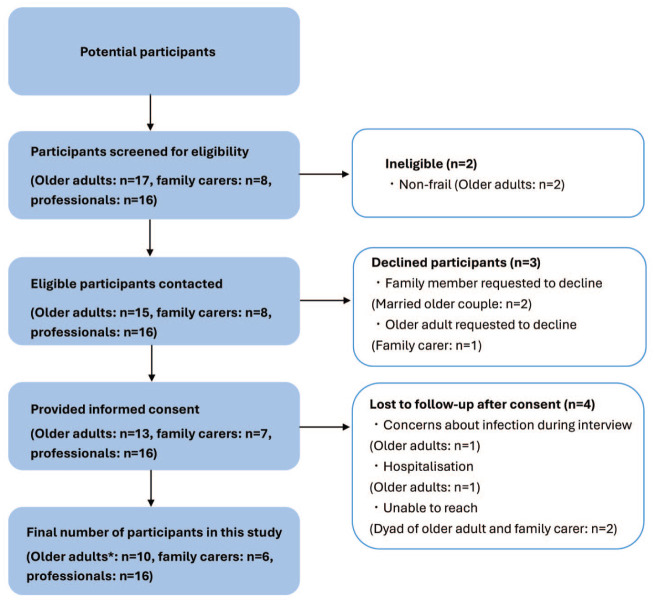
Participant recruitment flow chart. *One participant had dementia, requiring ongoing capacity assessment with the involvement of professionals and a family caregiver as consultee.

A total of 32 participants were recruited including older adults with frailty (*n* = 10), unpaid family caregivers (*n* = 6), homecare managers (*n* = 10), home-visit nurses (*n* = 3), and homecare physicians (*n* = 3) between April and July 2023 (Figure 1, recruitment flow chart and Table 3, participant characteristics). We conducted a total of 29 interviews, comprising 3 dyadic interviews (an older adult and a family caregiver) and 26 individual interviews. The mean interview duration was 72 min (range 49–132 min).

**Table 3.** Characteristics of participants.

Characteristics	*n*
Older adults with frailty (*n* = 10)	
Sex	
Male	4
Female	6
Age	
Median (range)	83 (70–96)
Japan Frailty Score*	
Median (range)	5 (4–8)
Living status	
Living with family members	8
Living alone	2
Family caregivers (*n* = 6)	
Sex	
Male	1
Female	5
Age	
Median (range)	61 (50–75)
Relationship with older adults	
Daughter	5
Son	1
Professionals (*n* = 16)	
Profession	
Care managers	10
Homecare doctors	3
Home-visiting nurses	3
Sex	
Male	5
Female	11
Years in current homecare role	
Median (range)	12 (3–26)

* Japan Frailty Score: ⩾4 pre-frailty or frailty.

### Themes

Three themes were generated from the participants’ experiences and perspectives on readiness for ACP. The themes and sub-themes are illustrated in Table 4 and Supplemental Appendix 3 (codes by respective theme). The themes illuminate the understanding of readiness for ACP among individuals and stakeholders and the influence of system-level factors.

**Table 4.** Themes and sub-themes of readiness for ACP.

Themes	Sub-themes
(1) ‘*Leaving decision-making to the family*’ and Bridging family dynamics	Trust-building relationships as foundations for ACP (*Opportunity*)
	The influence of relational authority and communication styles (*Opportunity*)
	Family involvement based on family relationship assessment and power dynamics (*Opportunit*y)
	Securing psychological safety of older adults (*Opportunity*)
	Acceptance of physical decline and delegation of decision-making (*Motivation*)
	The influence of older adults’ past experiences and ways of life (*Motivation*)
	Diverse forms of future preparation and awareness of ACP (*Motivation*)
(2) ‘*ACP is not part of our role’*: Diverging role expectations among professionals, and hesitation to engage in ACP	The impact of older adults’ limited physical and cognitive abilities (*Capabilit*y)
	Lack of knowledge and variations in understanding of ACP (*Capability*)
	Divergence in the perception of role expectations for professionals (*Motivation*)
	Reluctance to engage in ACP among older adults with frailty (*Motivation*)
(3) Transitional period: Social norms around death and dying, family structures, and health and social care systems	Challenges in collaboration due to hierarchy within multidisciplinary teams (*Mesosystem and Exosystem*)
	Limited health and social resources and constraints in information access (*Exosystem*)
	Absence of structured awareness initiatives for ACP (*Exosystem and Macrosystem*)
	Diversified perspectives and attitudes towards death and dying (*Macrosystem*)
	Diversified of family structures and family role expectations (*Macrosystem*)

#### Theme 1: ‘*Leaving decision-making to the family*’ and Bridging family dynamics

Older adults expressed ambivalence about the future, with a strong desire to maintain independence while recognising the inevitability of health decline and associated anxiety. Struggling to imagine their future, they preferred to focus on daily pleasures and available support to them in the present, rather than engage in future planning and were less ready to engage in ACP.

[Researcher: Does it feel like you’d rather not think too much about the future?]

I just go with the flow [. . .] I leave things up to how they turn out. I mean, there’s no point in pushing too much – it could cause trouble. We don’t know what’s going to happen, so saying unnecessary things or worrying too much doesn’t help either. (Older adult 8)

Older adults’ readiness for ACP was also shaped by their ways of life, including personal lifestyles and coping strategies. For instance, one older adult described a tendency to avoid sombre conversations and instead focus on the positive aspects of life – an attitude that influenced her approach to ACP.

Oh absolutely. I really hate [gloomy topics]. If [anything depressing] comes on TV, I turn it off right away or change the channel. (Older adult 4)

Conversely, older adults who regularly recorded daily life or planned ahead were more prepared to engage in ACP. Additionally, some older adults clearly communicated their needs to practitioners and family caregivers.

Some older adults clearly say, ‘I knew this would happen, so please handle it this way’. There are people like that. (Homecare manager 6)

Beyond individual-level factors, older adults’ readiness for ACP was shaped by their communication styles and the nature of their relationships. Both older adults and practitioners reported that older adults rarely shared their true feelings without a trusting relationship, particularly regarding personal matters.

[The primary care physician and homecare manager] are completely different. I can have casual chats with my homecare manager, but it’s not like that when I talk to the doctor. I can’t just ramble on like this, it’s harder to talk to that doctor. (Older adult 1)

In addition, older adults expressed a preference for discussing ACP with someone they trust, regardless of their professional background or whether they had medical expertise.

[The homecare manager] had medical experience [. . .] But because of that, I actually found it difficult to talk to her. It was like, ‘You’re fine, so there’s nothing to worry about’ – that kind of attitude. I really got the impression that she wasn’t very attentive, and I didn’t feel like I could consult her about much [. . .] I felt like I couldn’t have a proper, heartfelt conversation with her. (Family caregiver 4)

Older adults often communicated within the context of relationship. This influenced their readiness for ACP, and sometimes changed their preferences and wishes depending on whom they were speaking with.

The way the family and the patient speak and behave is completely different depending on whether they’re talking to a doctor or a nurse. (Homecare physician 2)

[My family caregiver] should understand without me having to say anything, though. (Older adult 12)

Especially, older adults’ readiness for ACP was particularly influenced by family dynamics, including preferences for involving not only family caregivers but also other family members, and concerns about maintaining harmony. In turn, family caregivers’ readiness was shaped by the older adults’ attitudes and circumstances. Differences of opinion and difficulties in open communication within families were common and were often influenced by the nature of family dynamics. Older adults often concealed their true feelings to maintain family harmony, fearing that disagreement could harm relationships with family caregivers, leading them to withhold their honest thoughts, which may hinder their readiness for ACP.

There are often differences of opinion [within the family]. We have to help align what the person wants for their life with what the family wants. (Homecare manager 3)

Practitioners noted this sometimes allowed the opinions of family members, including family caregivers, to override those of older adults, hindering their readiness for ACP.

They’re being considerate of their family – like watching their reactions, looking at their daughter-in-law’s face to see how she feels. Some people are thinking things through while keeping an eye on their family like that. (Homecare manager 2)

[To my daughter] If I make too many unreasonable requests, I feel like she might leave me be [. . .] I tell myself I have to hold back. Saying things like ‘I’m lonely’, or ‘Come more often’, or ‘Stay the night’. I feel like I shouldn’t ask for too much. (Older adult 8)

In contrast, some older adults believed their trusted family members would understand their wishes without explicit discussions. Having accepted physical deterioration and chosen to delegate decision-making to trusted family members, feeling confident that their choices would reflect their best interests and expressing a willingness to accept decisions made on their behalf.

It’s just easier to leave it to someone I trust, because I don’t have to think about anything. [Researcher: Like having preferences about what you’d want them to do. . .?] If I get my hopes up and they don’t do it. . . the disappointment would hit hard. I don’t need that kind of shock. (Older adult 4)

If I were to end up in that state, barely conscious, and my daughter decided on treatment, I would go along with it. [. . .] Even if those around me thought I looked pitiful and said, ‘Let her go’, I wouldn’t hold it against them. (Older adult 1)

Recognising these challenges and interrelated dynamics, all practitioners employed a ‘be a bridge’ approach, carefully observing these dynamics and working to uncover older adults’ concerns, to facilitate and enhance family communication, and to support their readiness for ACP. For instance, aiming to respect their wishes, prevent exclusion or distrust, and maintain harmony, homecare managers and home-visit nurses frequently first spoke with family caregivers before engaging with older adults, and also supported older adults in sharing their feelings with family caregivers when they felt it was necessary.

[Before speaking with the older adult], I make sure to properly ask the family caregiver about their understanding of ACP. Older adults often . . . well, I wouldn't say they’re weaker, but they can be in a more vulnerable position. If the focus is placed solely on the older adult, there’s a risk that the family caregiver might feel like the healthcare provider’s views have been imposed on the older adult. That’s why I make sure to understand the family caregiver’s perspective first. (Home-visit nurse 1)

#### Theme 2: ‘*ACP is not part of our role*’: Diverging role expectations among practitioners, and hesitation to engage in ACP

Although homecare physicians and home-visit nurses were not ready to engage in ACP with this population, they recognised ACP as one of their roles. In contrast, homecare managers were not ready to engage in ACP and were more likely to view ACP as a healthcare intervention and the responsibility to healthcare practitioners.

As a homecare manager, I feel that conversations about this kind of thing [ACP] are often seen as something for the medical side to handle. (Homecare manager 5)

On the other hand, all stakeholders recognised that homecare managers are often the most familiar with older adults and their families.

Our homecare manager knows about the home situation. She even knows things like, ‘Oh, my son is building a house here’, you know. (Family caregiver 5)

As part of our job, we always carefully assess older adults. [. . .] I believe many homecare managers know quite a lot about their past – in a way, their life journey. (Homecare manager 6)

A discrepancy in expectations regarding ACP was also observed among other stakeholders. Older adults and family caregivers expected health and social care practitioners to lead ACP discussions, while practitioners hesitated to initiate it, for various reasons.

I don’t think it [ACP] is something distant or irrelevant to older adults – it’s actually quite close to them. But I did feel that, when they’re faced with it [ACP], it can bring their mood down or feel emotionally heavy. (Homecare manager 2)

A key factor contributing to this hesitation was the perceived difficulty in identifying the ‘right time’ to initiate ACP. Identifying the optimal timing of ACP for older adults was perceived as challenging. While this timing was perceived as difficult to determine, health and social care practitioners generally considered that ACP should be conducted when older adults were physically and psychologically stable and that there was a ‘right time’ to engage in ACP.

Particularly, some homecare managers noted that when older adults were preoccupied with immediate daily concerns, their ACP readiness was limited. They emphasised that future care planning was unlikely to be productive until such concerns were addressed and that resolving these issues could serve as a foundation for building trust.

I often have [ACP] with older adults after we’ve dealt with the immediate issues. [. . .] For example, if someone is struggling with bathing, and they want that sorted right away. That becomes the first care plan – like, ‘Shall we arrange day care services or get a caregiver in?’ We take care of those things first [. . .] As things start to settle down, I would say something like, ‘You’re living home alone now, but have you thought about what happens when you can’t manage on your own, when things get more difficult?’ I often bring it up once the immediate, pressing issues have been dealt with [. . .] I feel like if we don’t solve the problems they’re facing right now, we can’t move on to the next step [. . .]. (Homecare manager 8)

Another reason for the difficulty in identifying the right timing was the uncertainty associated with frailty. Many practitioners reported a greater sense of readiness to engage in ACP with patients with cancer, whose illness trajectory is typically more predictable, compared to older adults with frailty, whose health trajectories were often uncertain.

I used to be very proactive about it [ACP] with cancer patients [. . .] Conversations with them often went more smoothly because many of them, and their families too, had already come to terms with the fact that everyone will eventually face death. (Homecare physician 3)

As a result, ACP was often delayed until triggered by marked deterioration in health or signs of approaching end-of-life. Some homecare physicians and home-visit nurses reported missed opportunities, as older adults had become unconscious or passed away before ACP discussions could occur.

The future is uncertain, and older adults with frailty often don’t have clear symptoms. I feel like [older adults] tend to see it as someone else’s issue, and [. . .] there’s often a strong hesitation to confront anything related to death. (Home-visit nurse 1)

Additionally, older adults’ physical and cognitive limitations, such as hearing difficulties, could influence all stakeholders’ readiness for ACP conversations. For instance, some older adults asked family caregivers to speak with health and social care practitioners on their behalf.

I can’t hear well, so [when the homecare manager comes] I ask my son to listen for me. (Older adult 5)

The family caregiver of older adult 5 observed that physical impairments often led practitioners to address them as the caregiver rather than the older adult, even when communication with the older adult was possible with assistance.

The homecare manager hasn’t really spoken much with my mother. It’s mostly been with me. (Family caregiver 4)

Furthermore, a lack of knowledge and varied interpretation of ACP also hindered readiness for ACP among all stakeholders. Health and social care practitioners often viewed ACP narrowly, focusing on end-of-life discussions, medical and care planning, or as a tick-box exercise for documenting treatment preferences.

When it comes to what we ask during [ACP], it’s mainly about how to respond during periods of sudden deterioration. (Homecare physician 2)

People tend to start thinking about it more seriously only when someone has only a little time left [their life] or when their condition is unlikely to improve. (Homecare manager 5)

Some older adults also stated that such discussions were premature for them, as they perceived themselves as too well to consider this topic and still independent.

I’ve never really had those kinds of conversations before, and since I feel fine now, I don’t feel like I need to ask or think about that sort of thing [ACP]. (Older adult 6)

Those who perceived that they had engaged in ACP primarily focused on expressing their preference to avoid life-prolonging treatments and their preferred place of death, with little emphasis on the process leading to these decisions or conversations on ‘what matters most to them’. Many had not engaged in ACP conversations but were preparing for the future in other ways, such as decluttering belongings and managing inheritance arrangements.

I’ve put things like my pension certificate and life insurance documents together in a drawer. [Researcher: Have you told your daughter they’re there?] Yes, I’ve told her – ‘They’re in here’. (Older adult 12)

#### Theme 3: Transitional period: Social norms around death and dying, family structures, and health and social care systems

The interviews revealed that social norms surrounding death, dying, and family structures were experienced as increasingly diverse. Participants’ attitudes varied, with some open to discussing these topics while others were hesitant.

The older adults in my care tend to use the word ‘death’ more often. They’ll say things like, ‘When I die, I want this to happen’. I feel like, at that stage in life, it feels more familiar to them. Death isn’t necessarily right around the corner, but they’re at an age where they start to think about it. [. . .] So, I think there might be a gap between us feeling like it’s inappropriate or disrespectful to bring up death in conversation, and how they actually perceive it. [. . .] Of course, it depends on the person and their age. (Homecare manager 10)

Family role expectations also differed: some older adults lived with or near their adult children for support, while others preferred independent living, relying on professional care. Notably, resistance to seeking professional care is decreasing. At the same time, practitioners noted difficulties in finding time to discuss care matters with family caregivers, especially when family lived separately or, despite cohabiting, were often unavailable due to work commitments during the daytime when homecare managers visited.

Ideally, I’d like to be cared for at home. I’d want professional carers like homecare workers to support me. That’s because having practitioners would mean less of a burden on my family. (Older adult 2)

Hierarchical barriers within multidisciplinary teams were evident, with homecare managers often facing challenges in communicating with healthcare practitioners, particularly physicians. Despite having valuable information to share, many felt hesitant to engage directly.

The majority of homecare managers have a background in caregiving – I’m one of them. [. . .] I also believe many of us feel anxious about communicating with healthcare practitioners, but we still want to engage with them because we know it’s essential. We just lack confidence, and that holds us back. [. . .] I guess, in some way, there’s still a sense of discomfort when interacting with healthcare practitioners. (Homecare manager 9)

A complicated health and social care system was also identified as a barrier to readiness for ACP by family caregivers. In particular, the Long-Term Care Insurance System and other related services were perceived as difficult to understand, varying between municipalities, and subject to frequent revisions. These complexities made it challenging for families to navigate the system and anticipate the support available in the future.

Yes, exactly. If there were something like a summary with pictures or a chart [about homecare services or long-term care insurance], I think that alone would probably help people decide – like, which to choose: a care facility or [homecare] [. . .] I think there are a lot of people [making decisions without that kind of information]. Probably. I felt the same way too. If my mum hadn’t said she wanted to try [homecare], I would’ve thought it was impossible and just decided she should go [into a care facility]. (Family caregiver 4)

Moreover, older adults and their family caregivers often found ACP discussions abstract and challenging, emphasising the need for health and social care practitioners’ guidance. Similarly, practitioners expressed uncertainty about conducting ACP, highlighting the need for clear guidelines, training, and supporting materials to empower their readiness for ACP.

When someone asks us [older adult and family carer], ‘[What do you think] about the future?’ I’m like, About what? [. . .] It [ACP] feels too vague, so I’d like a practitioner – or even Ms. A [our homecare manager] – to come and help. Like, ‘In other homes, they’ve done this or talked about that’, something like a hint to help us connect the dots. If you’re suddenly asked, ‘What do you think [about future]?’ it’s natural to say, ‘I haven’t thought about it’. (Family caregiver 3)

[When it comes to ACP], I’m not really sure exactly what we should discuss or how far we should go – like, there’s no clear model to follow. [. . .] I feel like I’d really like to have some kind of template or materials. If there were a guide that says something like, ‘You might want to ask about these things’ – they could be useful later, I’d want that. (Homecare manager 8)

## Discussion

### Main findings of the study

Readiness for ACP in community-dwelling older adults with frailty was influenced and shaped by individual- and system-level factors, as well as their interactions, and followed a dynamic, continuous process. These included an individual’s communication style and coping approaches, such as how they dealt with challenges and with whom they communicated. Importantly, system-level factors appeared to exert an even greater influence, including the readiness of family caregivers and health and social care practitioners, hierarchical dynamics among practitioners, and the transitional phase of social norms around death and dying, and evolving family structures.

**Figure 2.**
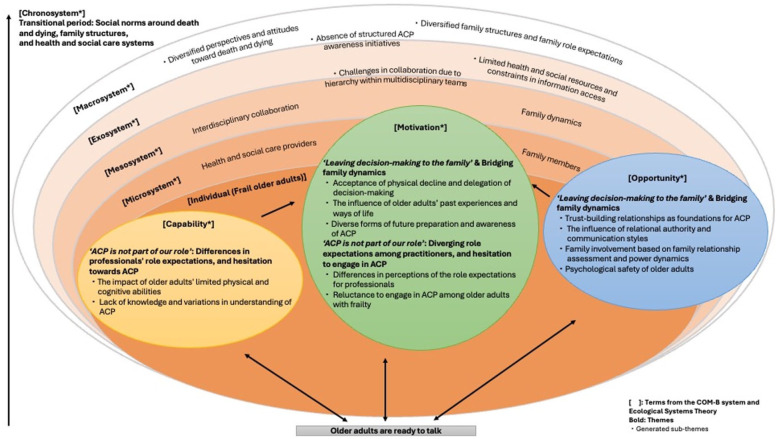
Conceptual model of readiness for ACP among community-dwelling older adults with frailty. *The three circles represent *capability, opportunity*, and *motivation*, based on the COM-B system,^36^ with arrows indicating interrelationships. Their size reflects broader influences beyond the individual. Surrounding layers are adapted from Ecological Systems Theory^37^: microsystem, *mesosystem, exosystem*, and *macrosystem*. These layers interact dynamically and shape older adults’ readiness for ACP. The *chronosystem* is shown outside the circles, as it represents the influence of time and sociocultural transitions across all factors.

The dynamic interplay of individual- and system-level factors shaping ACP readiness is illustrated in the conceptual model (Figure 2). The model is informed by two underpinning theoretical frameworks: the COM-B system^36^ and the Ecological Systems Theory.^37^ The conditions within the model are interconnected, reflecting the ongoing interaction between individual and systemic influences, as guided by the underlying theoretical principles.

Conditions informed by the COM-B system include factors related to older adults, as well as individual-level factors related to family caregivers and health and social care practitioners. The *capability*, such as older adults’ limited physical and psychological abilities and lack of knowledge about ACP, and the *opportunity*, such as family dynamics and the relationships with practitioners, may influence the *motivation*, thereby shaping individuals’ readiness for ACP.

Beyond individual behaviour change, additional factors were illustrated using the Ecological Systems Theory. In this model, family caregivers and practitioners are situated at the *microsystem* and *mesosystem* levels, as their readiness for ACP influences that of older adults within the broader system. The *exosystem* relates to structures within health and social care systems, while the *macrosystem* encompasses broader societal norms. The *chronosystem* is conceptualised as spanning all levels, from individual to system-wide. It includes temporal and transitional factors such as changing social norms around death and dying, evolving family structures, and the uncertain health trajectories of older adults with frailty.

### What this study adds

Older adults with frailty tended to prioritise day-to-day life over planning for the future, often considering ACP as irrelevant. This finding aligns with previous studies from other countries^50,58^ and may represent a general pattern among this population. Therefore, it is essential to integrate current and adaptive care planning within a broader, continuous framework^15,59^. Rather than focusing solely on the traditional biomedical emphasis on future medical care planning, this approach prioritises understanding ‘what matters to the person’ in both current and future care.^16,50[Bibr bibr49-26323524251395654]^

However, across participant groups, knowledge of ACP was limited and often biased. This was frequently focused on biomedical decision-making about withholding or withdrawing futile care at the end-of-ife, such as cardiopulmonary resuscitation as commonly reported in the literature.^60^ In line with these perceptions, older adults with frailty were often regarded as premature candidates for ACP compared with other population groups.^14,21^ Such a narrow perspective may have hindered individuals’ readiness for ACP, particularly by limiting their *capability* as defined in the COM-B system. Furthermore, this phenomenon can be understood through the COM-B system, which emphasises how the *capability* (Sub-theme: Lack of knowledge and variations in understanding of ACP) and shape the *motivation* (Sub-theme: Reluctance to engage in ACP among older adults with frailty). For instance, homecare physicians and home-visit nurses tended to postpone discussions, recognising that ACP conversations often take place at points of deterioration or as signals of impending death, by which time the person is often too unwell to discuss or plan their priorities for care.^21^ Homecare managers also perceived ACP as discussion about medical care planning at the end of life, and as the responsibility of healthcare practitioners specialising in medical care, which aligns with findings from a previous study.^61^ This perception may have reinforced the view that ‘*ACP is not part of our role*’.

From the perspective of older adults, difficulty imagining their end-of-life stage, may also have contributed to the perspective expressed by older adults: ‘*Leaving decision-making to the family*’, rather than sharing what matters to them. Another possible explanation for this tendency may be the cultural emphasis on relational authority in Japan, as identified in previous studies,^62,63^ where decision-making is often entrusted to trusted family members. While cultural factors may play a role, this tendency is not solely attributable to them. It may also reflect a broader pattern observed among older adults across a range of conditions^64^ and care settings^65,66^ internationally, in which they let go of control over their lives^64^ and delegate decision-making to someone the trust. Older adults frequently prepared for the future in various ways, such as inheritance planning, even if they had not engaged in ACP. This is consistent with reports heterogeneous readiness depending on the specific conditions of future care planning.^54,58^ These preparations frequently occur without the involvement of healthcare practitioners, as shown in previous studies conducted with broader populations.^18 [Bibr bibr59-26323524251395654]^ Therefore, healthcare-focused perspectives alone are insufficient for ACP, suggesting a need to include a broader lends, such as social care perspectives.

In the context of homecare, homecare managers were recognised by all stakeholders as key practitioners in supporting and initiating the ACP process. Their unique position stems from close, long-term relationships with older adults, a deep understanding of family dynamics identified in this study, and their ability to provide support from a lifestyle perspective.^68,69^ The significant role of homecare managers in ACP is further highlighted by the emphasis older adults placed on trusting relationships, both in this study and in previous research.^58,70^ Incorporating the perspectives of homecare managers in ACP processes could help to understand and support older adults’ readiness for ACP.

However, homecare managers reported that hierarchical structures hindered communication with healthcare practitioners; a point echoed in previous research.^71^ This limited interprofessional collaboration with healthcare practitioners, with homecare managers struggling to convey the concerns they had observed. This finding is in line with a survey conducted in Japan, which found that 50% of homecare managers faced challenges in communicating with physicians.^72^ There is no formal clinical training and support for them,^29^ and they are often isolated from access to healthcare practitioners.^28^ Therefore, to enhance homecare managers’ readiness for ACP, it is important not only to strengthen the ‘capability’ (Sub-theme: Lack of knowledge and variations in understanding of ACP) but also to adopt a system-based approach. In particular, addressing this hierarchy is crucial for fostering open communication among health and social care practitioners and integrated working to empower homecare managers’ readiness to engage in ACP conversations.

Family roles in the care of older adults were diverse, ranging from co-residing or nearby children providing regular support to older adults preferring independent living supported by paid formal care. This may reflect an growing variation in the role of family caregivers in recent years in Japan^70^ where traditionally, relying on paid formal care was perceived as children neglecting their familial duties.^70,71^ As noted in previous research, increased female employment^11^ and the introduction of the Long-Term Care Insurance System have played a significant role in decreasing reliance on family caregivers and in normalising the utilisation of paid formal care. These changing social norms, including evolving attitudes toward family responsibility, may shape how older adults perceive and approach ACP. This underscores the need for open dialogue rather than relying solely on family responsibility. This could partly explain variations in ACP readiness.

### Strengths and limitations of the study

A strength of this study is its exploration of readiness for ACP from various stakeholders, combined with a theory-based analysis. Specifically, it includes homecare managers, who are social care practitioners, whose perspectives have often been underrepresented. These approaches provided a comprehensive and systematic understanding of older adults’ readiness for ACP, which could contribute to the development of interventions to empower older adults with frailty in this process.

However, the study is limited by the underrepresentation of participants with severe frailty. Male perspectives were also limited, with only one of the six family caregivers being male. This may reflect traditional gender roles, which often position women as primary caregivers.^70^ Further research is needed to explore more diverse perspectives on readiness for ACP.

### Implications for clinical practice and research

Beyond individual-level factors, various relational, systemic, and societal-level factors influencing ACP readiness were reported. As a result, the most beneficial interventions to enhance older adults’ readiness for ACP are likely to vary depending on these factors. In particular, hierarchical structures within and across health and social care practitioner groups were identified as barriers to an interdisciplinary approach to ACP. Overcoming these barriers requires integrated working between health and social care practitioners, which is especially important for empowering homecare managers, who know older adults well, to provide opportunities for individuals to consider and plan their future care. For instance, joint ACP training across professions may foster shared understanding, and common ACP goals may deepen mutual understanding and enhance communication among these practitioners, as suggested by previous work on multidisciplinary teams in Japan.^72^

To empower older adults’ readiness for ACP, enhancing the readiness of health and social care practitioners is critical. In particular, training is needed to help practitioners address the barriers they have identified as hindering ACP readiness. This includes expanding their understanding of ACP beyond the biomedical focus to encompass a broader concept, and developing communication skills such as creating a psychologically safe space and introducing ACP in a sensitive manner. Specifically, guidance and support for social care practitioners should be prioritised as they are often less familiar with ACP, which has traditionally emerged from healthcare-led interventions. Training should aim to enhance practitioners’ competence and confidence in ACP, help them prioritise it in practice, and enable them to assess and strengthen older adults’ readiness.

For older adults, information related to ACP should be provided in lay language, with consideration for their limited physical and cognitive capabilities. Moreover, such information may be more beneficial if provided by trusted practitioners who know them well through regular contact over time. Older adults reported the importance of such trusted relationships in ACP given the sensitive nature of these conversations and may expect follow-up discussions. To enhance older adults’ readiness, conversations are best initiated with a focus on current concerns and what matters to them now. These topics are familiar and important to older adults, and are best discussed with a trusted facilitator. Importantly, readiness can vary and change over time, requiring a person-centred approach and avoidance of assumptions such as that older adults are unwilling to engage or not capable to do so. This underscores the need for case-by-case assessment that considers not only the individual but also family involvement. The role of the family needs to reflect the family’s power balance, as they are often key stakeholders in enhancing older adults’ readiness for ACP.

Future research should include the development and evaluation of interventions to enhance older adults’ readiness for ACP, informed by the findings of this study. In designing such interventions, relational, systemic and societal contexts such as interdisciplinary collaboration should be considered, and a system-wide approach should be adopted to reflect these contextual influences.

## Conclusion

This study offers new insights into the contextual and systemic influences shaping readiness for ACP among older adults with frailty in Japan, drawing on perspectives from older adults, family caregivers, and health and social care practitioners. The findings demonstrate that readiness is not a fixed individual attribute but a dynamic, relational process shaped by interactions with family caregivers, health and social care practitioners, and the wider systemic and societal environment. Together, the COM-B system and Ecological Systems Theory together provide a comprehensive framework for guiding context-sensitive, relationship-based interventions to support ACP readiness in practice. Enhancing ACP readiness, therefore, requires a system-wide approach. This includes addressing hierarchical barriers to promote interdisciplinary collaboration among health and social care practitioners, recognising the vital yet often overlooked role of homecare managers, involving families, and adapting approaches to the local context, including the health and social care systems and prevailing social norms.

## Supplemental Material

sj-docx-2-pcr-10.1177_26323524251395654 – Supplemental material for Exploring readiness for advance care planning in Japan: A qualitative interview study with older adults with frailty, family caregivers, and health and social care practitioners in the homecare setting Supplemental material, sj-docx-2-pcr-10.1177_26323524251395654 for Exploring readiness for advance care planning in Japan: A qualitative interview study with older adults with frailty, family caregivers, and health and social care practitioners in the homecare setting by Miki Fujimoto, Jonathan Koffman, Ito Nagata, Yukihiro Sakaguchi and Catherine J. Evans in Palliative Care and Social Practice

sj-pdf-1-pcr-10.1177_26323524251395654 – Supplemental material for Exploring readiness for advance care planning in Japan: A qualitative interview study with older adults with frailty, family caregivers, and health and social care practitioners in the homecare setting Supplemental material, sj-pdf-1-pcr-10.1177_26323524251395654 for Exploring readiness for advance care planning in Japan: A qualitative interview study with older adults with frailty, family caregivers, and health and social care practitioners in the homecare setting by Miki Fujimoto, Jonathan Koffman, Ito Nagata, Yukihiro Sakaguchi and Catherine J. Evans in Palliative Care and Social Practice
