# Supplementary material for: Exploring readiness for advance care planning in Japan: A qualitative interview study with older adults with frailty, family caregivers, and health and social care practitioners in the homecare setting
Source: Palliat Care Soc Pract. 2025 Nov 28;19:26323524251395654. doi: 10.1177/26323524251395654 (PMC12665015; doi:10.1177/26323524251395654)
Supplement: sj-docx-2-pcr-10.1177_26323524251395654 – Supplemental material for Exploring readiness for advance care planning in Japan: A qualitative interview study with older adults with frailty, family caregivers, and health and social care practitioners in the homecare setting [file sj-docx-2-pcr-10.1177_26323524251395654.docx]

**Appendix 1: Reflexive Thematic Analysis Reporting Guidelines (RTARG) checklist**

| **Guideline Items** | **How it was addressed** |
| --- | --- |
| **1. The Introduction** |  |
| **1.1 Background and rationale** |  |
| 1.1.1. Provide a robust context and rationale for the proposed research in the Introduction. | A robust rationale was provided through discussions of relevant research and theories, along with a description of the context of the research site in **1. Background**. |
| 1.1.2. Clearly articulate a research question – one that is methodologically coherent. | The research aims and objectives were aligned with a qualitative methodology, which allows for in-depth exploration of participants' experiences and perspectives on readiness for ACP. |
| **1.2 “Owning your perspectives”** |  |
| 1.2.1. Include information on guiding theoretical assumptions and other (e.g., explanatory) theory informing the use of TA. | This study was guided by a pragmatic approach, which supports the use of reflexive thematic analysis to flexibly explore real-world experiences and practical implications. In line with pragmatism, the research question is prioritised over specific methods or paradigms (Tashakkori and Teddlie, 2010), allowing for a focus on meaningful, actionable insights into readiness for ACP. The study is also grounded in the COM-B system (Michie et al., 2011), which provides a framework for understanding individual behaviour change mechanisms, and Ecological Systems Theory (Bronfenbrenner, 1994), which situates readiness within broader social and environmental contexts. These frameworks informed every stage of the research process, from shaping the research aims to guiding data collection and analysis. By integrating these theories, the study offers a comprehensive understanding of the factors influencing readiness for ACP, aligned with its pragmatic goal of generating actionable and contextually relevant insights. |
| 1.2.2. Report in a way that is consistent with stated theoretical assumptions throughout. | The report maintains theoretical coherence by consistently applying language and concepts aligned with reflexive thematic analysis. Research subjectivity was acknowledged through the use of field notes, reflexive journaling, and self-positioning statements, which enabled critical engagement with the data and enhanced interpretative transparency by recognising the researcher’s influence on theme development and data interpretation. |
| 1.2.3. Evidence methodological coherence/integrity in both the research and the report. | Methodological coherence was achieved by aligning the theoretical assumptions, research aim, methods of data generation, and the use of reflexive thematic analysis within a pragmatic framework. |

| **(Continued) Appendix 1** |  |
| --- | --- |
| **Guideline Items** | **How it was addressed** |
| 1.2.4. Show evidence of reflexive practice. | In our study, reflexive practice was evidenced through a critical examination of the researchers' professional and personal positioning in relation to the research topic and participant group, as outlined in this table. For instance, the primary researcher (MF), a Japanese national with a nursing background, drew on personal experiences in oncology nursing at an acute tertiary care hospital and over five years of research in ACP, including questionnaire surveys and interview studies. These experiences informed the research focus and shaped the interpretative lens throughout the research process. Reflexivity was further enhanced through collaboration with researchers from diverse backgrounds: IN, a Certified Nurse Specialist in Homecare Nursing with over 10 years of experience as a home-visit nurse at the research site; YS, a psychology professor specialising in bereavement care; and CJE and JK, British professors specialising in palliative care and qualitative research.  Field notes and reflexive journaling were employed throughout the research process to identify and critically reflect on how the researchers' perspectives shaped the study. These practices enabled the team to examine potential influences of our positioning on data interpretation and analysis. Reflexive practices are explicitly integrated into the research narrative and further detailed in this table. |
| 1.2.5. Write in a methodologically coherent style. | A third-person writing style was adopted in line with common practices in the journal to ensure clarity and reader-friendliness. However, the manuscript was carefully crafted to incorporate language and concepts relevant to reflexive thematic analysis, ensuring alignment with its principles and avoiding a positivist tone. |
| **2. The Methodology** |  |
| **2.1. Participants/data items** |  |
| 2.1.1. Describe selection of participants/data items. | It was demonstrated in **2.3 Setting and Participants**, and **2.4 Selection of participants.** |
| 2.1.2. Describe number of participants/data items; provide a rationale or explanation around dataset or participant group size/composition. | It was detailed in **2.4 Selection of participants**. Individual interviews with professionals were conducted, as MF anticipated that some might not feel safe sharing their honest views due to professional hierarchies, based on MF's clinical experience and the stakeholder consultation. |
| 2.1.3. Discuss characteristics of participants/data items. | It was provided participants' demographic backgrounds in **Table 3: Characteristics of Participants.** However, anonymity was ensured by presenting aggregated information and omitting specific identifiers where appropriate. |
| 2.1.4. Detail ethical approval and ethical code/principles followed, participant informed consent, etc. | It was described it in **2.8 Ethical approval.** |
| **(Continued) Appendix 1** |  |
| **Guideline Items** | **How it was addressed** |
| **3. Dataset generation** |  |
| 3.1. Provide some rationale for method(s) for data generation/data item sources  chosen. | Multiple stakeholders were included to gain a comprehensive and in-depth understanding of readiness for ACP. This approach aligns with the research aim and objectives, which focus on understanding the experiences and perspectives related to ACP readiness in older adults with frailty, a process that is inherently dynamic. The COM-B system (Michie et al., 2011) and the Ecological Systems Theory (Bronfenbrenner, 1994) were used to guide the exploration, providing theoretical frameworks to understand the interplay of individual capabilities, opportunities, motivations, and broader contextual factors. |
| 3.2. Describe development and/or characteristics of data generation tool(s). | We used a topic guide and explained how it was developed in **2.6 Data collection.** |
| 3.3. Include details such as modality and/or setting of data generation, time frame, and other pertinent procedural information. | Please refer to **2.6 Data collection**. |
| 3.4. Describe who conducted any interactive data generation (which author or research role), and how. | All interviews were conducted by MF, a Japanese female with clinical experience as a nurse and skills in qualitative interviewing. MF’s background provided insight into the clinical context, facilitating rapport-building and an empathetic understanding of participants' perspectives. As MF had no prior relationship with the participants, she took time to carefully explain the research purpose and used informal conversations to build rapport. The interviews were conducted in Japanese, audio-recorded, and transcribed in Japanese. Throughout the interviews, MF maintained field notes and engaged in reflexive journaling, capturing both verbal and non-verbal communication with stakeholders and participants from recruitment to completion. MF transcribed the interview data in Japanese and read the transcripts multiple times to gain a thorough understanding of participants' experiences and perceived readiness for ACP. Additionally, she reviewed field notes and reflexive journals, adding further reflections during the analysis process. |
| 3.5 Describe the size/scope of dataset and dataset items. | It is reported in **3.1 participants.** |
| 3.6 Describe, and if relevant explain, any preparation of data for analysis. | Audio data from the interviews were transcribed verbatim in Japanese, maintaining the original language to preserve meaning and context. Minor typographical errors were corrected for readability, ensuring clarity without altering participants' intended meaning. Pseudonyms were assigned to each participant, and these were used consistently throughout the analysis and reporting to maintain confidentiality while allowing for clear participant referencing. |

| **(Continued) Appendix 1** |  |
| --- | --- |
| **Guideline Items** | **How it was addressed** |
| **4. Data analysis** |  |
| 4.1. Provide some rationale for use of RTA, and, where relevant, for combining RTA with other approaches and procedures. | Reflexive Thematic Analysis is well suited for capturing the depth and complexity of subjective experiences and perspectives on readiness for ACP. Its flexibility enables researchers to engage deeply with participants' narratives, allowing for an understanding of the nuanced and dynamic factors influencing readiness for ACP. |
| 4.2. Describe specific orientation to RTA. | It was detailed in **2.7 Data analysis.** |
| 4.3. Discuss how the researcher(s) engaged with the analytic process. | It was detailed in **2.7 Data analysis.** |
| 4.4. Where more than one person is involved, describe who analysed the data (author or research role). | Differences in data interpretation or coding were resolved through reflexive discussions among the researchers, ensuring that the themes reflected diverse viewpoints and captured the complexity of readiness for ACP. This collaborative process adhered to the principles of reflexivity, recognising the influence of the researchers’ backgrounds and perspectives on the analysis. |
| 4.5. Use language to describe the process and products of RTA that is coherent with the values and assumptions of RTA. | It was carefully reported using language aligned with the values and assumptions of Reflexive Thematic Analysis. |
| **5. The Analysis** |  |
| **5.1. Reporting the data analysis** |  |
| 5.1.1. Provide an overview of themes or thematic structure. | An overview of all themes and sub-themes was provided in **Table 4**, along with the codes by respective theme in **Appendix 3**. |
| 5.1.2. Ensure theme conceptualisation is appropriate to RTA, and any divergences are justified and explained. | It was detailed in **2.7 Data analysis.** Themes were carefully generated and named to ensure they encapsulate shared meaning, organised around a central organising concept, as recommended in the guidelines. Each theme reflects a deeper level of interpretation, going beyond mere topic summaries. In generating the themes, we focused on capturing patterns of meaning that resonate across the dataset, ensuring they are conceptually coherent and distinct from one another through repeated discussions among researchers. |
| 5.1.3. Name themes appropriately. | It was detailed in **2.7 Data analysis.** Themes were named to capture their essence and overarching narrative, moving beyond simple topic identification. Simplistic theme names were avoided, with an emphasis on reflecting the deeper narrative uncovered during analysis and incorporating quotations where appropriate. |

| **(Continued) Appendix 1** |  |
| --- | --- |
| **Guideline Items** | **How it was addressed** |
| 5.1.4. Report themes in sufficient depth and detail. | Themes were reported with depth and detail in **3.2 Themes**, ensuring they were multifaceted and included both data and analytic narrative, in accordance with the recommendations of reflexive thematic analysis. |
| 5.1.5. Use subtheme judiciously. | Details of how they were developed them are provided in **2.7 Data analysis** about how we developed them. Furthermore, detailed descriptions of the sub-themes are provided in **3.2 Themes.** |
| 5.1.6. Ensure the analytic narrative explains the meaning and significance of the data. | The analytic narrative for each theme was developed to explain its meaning and significance in relation to the topic, research question, and dataset. Each theme was supported by an interpretative narrative that highlighted underlying patterns of meaning. The overall story of the findings, including the positioning of each theme and sub-theme, are presented in **3.2 Themes.** |
| 5.1.7. Provide an appropriate balance of analytic narrative and data extracts – both data extracts and analytic narrative matter. | The analytic narrative and data extracts were balanced to avoid mere narrative summaries, with a focus on demonstrating sufficient and meaningful analytic interpretation. |
| 5.1.8. Demonstrate coherence between analytic narrative and illustrative/evidentiary data extracts. | A clear connection between the analytic narrative and the data extracts was established by selecting examples that strongly supported the claims. Each data extract was explained within the narrative to clarify its importance and relevance. To enhance the credibility and depth of the analysis, alternative interpretations of the data were also considered. |
| 5.1.9. Integrate existing research and theory into the analytic narrative. | Existing research and theoretical frameworks were integrated into the analytic narrative to provide context and enrich the interpretation of themes. The COM-B system (Michie et al., 2011) and the Ecological Systems Theory (Bronfenbrenner, 1994) were specifically used as theoretical underpinnings to frame the analysis and interpret the findings. These frameworks contextualised the patterns of meaning within the data, offering a deeper understanding of readiness for ACP by considering both individual behaviour change and the influence of external factors. Relevant previous studies were also referenced to enhance the interpretative analytic narrative and reflect contextualised meaning. This was reported in **4. Discussion**, in line with common practices in the journal to ensure clarity and reader-friendliness. |
| **6. The Final Section – A General Discussion or “Conclusions”** |  |
| **6.1. Quality, evaluation and conclusions** |  |
| 6.1.2. Draw analytic conclusions across themes. | The manuscript ensures that analytic conclusions are drawn across the themes, highlighting the overarching narrative of the analysis in **4. Discussion**. |

| **(Continued) Appendix 1** |  |
| --- | --- |
| **Guideline Items** | **How it was addressed** |
| 6.1.3. Discuss implications or directions for future research. | It was detailed in **4.4. Implications for clinical practice and research** section. |
| 6.1.4. Use and report quality practices coherent with RTA. | The study employed quality practices aligned with the principles of Reflexive Thematic Analysis to ensure rigour and depth in the analysis. Field notes and reflexive journaling were used throughout the research process to document evolving thoughts and interpretations, enhancing reflexivity rather than seeking consensus or validation. Critical discussions with other researchers provided alternative perspectives, deepening insights and refining the analysis. In reporting, language relevant to Reflexive Thematic Analysis was applied. |
| 6.1.5. Evaluate the research from a Big Q standpoint. | The study employed quality practices aligned with the principles of Reflexive Thematic Analysis to ensure rigour and depth in the analysis. Field notes and reflexive journaling were used throughout the research process to document evolving thoughts and interpretations, enhancing reflexivity rather than seeking consensus or validation. The characteristics and context of the participant groups, along with the data collection methods, were recognised as integral to shaping the findings, rather than limitations. Critical discussions with other researchers provided alternative perspectives, deepening insights and refining the analysis. In reporting, language consistent with Reflexive Thematic Analysis was applied to reflect the situated nature of the study. |
| 6.1.6. Include reflections on research process and practices, including researcher  reflexivity. | Researchers' reflections were incorporated into the critical discussion, highlighting how our positionality informed the analytic lens and the construction of themes presented in this table. |

**Appendix 2: Topic guides**

**Topic guide (Older adults)**

*Thank you very much for taking the time to speak with me today.*

*The length of the interview would be approximately 60 mins.*

*If you feel unwell, or if you want to stop the interview, please do let me know. You do not have to give a reason.*

*First of all, can I ask a few questions about you?*

**1. Personal information**

1) May I ask your age?

2) May I ask if you have any current health conditions?

3) May I ask about your family or close friend who you can turn to for support if needed?

Who do you live with or do you live by yourself?

*During this interview, I’d like to ask you some questions for you. There is no right or wrong answer to any of my questions. So please share your honest opinion with me. Are you ready to start?*

**2. Explore understanding of advance care planning (ACP)**

1) Have you ever heard of the term **Life conference**, **advance care planning**, **advance directives**, and **living will**?

***Life Conference** is the name created by the Ministry of Health, Labour and Welfare as an interpretation of ACP, aimed at making it more understandable for the general public.

2) What kind of information and knowledge do you have related to ACP?

3) Share the definition of ACP below.

***What is Life Conference (Advance care planning)?**

“An individual **thinking** and **discussing** with his or her **family** and other people close to them with the support as necessary of **healthcare providers** who have established a **trusting relationship** with the individual **concerning preparations for the future**: his or her **current state of health and future way of life** and **medical treatment and care** that the individual wishes to receive in the future (Miyashita et al, 2022)”

*Ministry of health, Labour and Welfare named ACP as Life Conference to make it more familiar to the public.

**3. Perspectives and experience with ACP**

1) What do you think about **Life Conference (Advance care planning)**? Why?

2) Have you ever had conversations related to **Life Conference (Advance care planning)** with family members or professionals?

-> If so, what did you discuss? with whom? When? Why?

-> If not, Why not?

**4. Facilitators and barriers on ACP (COM-B system & Ecological Systems Theory)**

1) What would enable to be ready to talk about **Life Conference (Advance care planning)**?

2) What could prevent some being able to engage in **Life Conference (Advance care planning)**?

-> How do you think we can minimise these obstacles?

**Questions will be given based on COM-B model (capability, opportunity, and motivation) and Ecological Systems Theory (microsystem, mesosystem, exosystem, macrosystem and chronosystem)*

**5. Needs on ACP (COM-B system & Ecological Systems Theory)**

1) What kind of practical support do you need, or did you get by health and social care practitioners to be ready to talk about **Life Conference (Advance care planning)**?

2) What kind of practical support do you need, or did you get from other aspects (e.g. family, community support, healthcare systems, policy or other social care systems) to be ready to talk about **Life Conference (Advance care planning)**?

3) Who do you think should initiate the Life Conference?

**Questions will be given based on COM-B model (capability, opportunity, and motivation) and Ecological Systems Theory (microsystem, mesosystem, exosystem, macro system and chronosystem)*

**6. Conclusion questions**

1) Is there anything you would like to share?

2) Do you have any questions?

*That concludes our interview.*

*Thank you very much for taking the time to speak with me today.*

*If you have any further questions or need clarification, please do not hesitate to contact me using the details provided in the participant information sheet.*

**Throughout the interview, the term ACP would be changed depending on the participants’ understanding. (e.g. Life Conference, having conversations about your preferences and wishes on current and future care and treatment and others)*

**Topic guide (Family carers)**

*Thank you very much for taking the time to speak with me today.*

*The length of the interview would be approximately 60 mins.*

*If you feel unwell, or if you want to stop the interview, please do let me know. You do not have to give a reason.*

*First of all, can I ask a few questions about you?*

**1. Personal information**

1) May I ask your age and the age of your family member (the older adult)?

2) May I ask what your relationship is with your family member (the older adult)? Do you live together?

3) May I ask if your family member (the older adult) have any current health conditions?

4) May I ask about who provide support to your family member (the older adult) when needed?

*During this interview, I’d like to ask you some questions for you. There is no right or wrong answer to any of my questions. So please share your honest opinion with me. Are you ready to start?*

**2. Explore understanding of advance care planning (ACP)**

1) Have you ever heard of the term **Life conference**, **advance care planning**, **advance directives**, and **living will**?

***Life Conference** is the name created by the Ministry of Health, Labour and Welfare as an interpretation of ACP, aimed at making it more understandable for the general public.

2) What kind of information and knowledge do you have related to ACP?

3) Share the definition of ACP below.

***What is Life Conference (Advance care planning)?**

“An individual **thinking** and **discussing** with his or her **family** and other people close to them with the support as necessary of **healthcare providers** who have established a **trusting relationship** with the individual **concerning preparations for the future**: his or her **current state of health and future way of life** and **medical treatment and care** that the individual wishes to receive in the future (Miyashita et al, 2022)”

*Ministry of health, Labour and Welfare named ACP as Life Conference to make it more familiar to the public.

**3. Perspectives and experience with ACP**

1) What do you think about **Life Conference (Advance care planning)**as a family member? Why?

2) Have you ever had conversations related to **Life Conference (Advance care planning)**?

-> If so, what did you discuss? with whom? When? Why?

-> If not, Why?

**4. Facilitators and barriers on ACP (COM-B system & Ecological Systems Theory)**

1) What would enable to be ready to talk about **Life Conference (Advance care planning)** for you and your family member?

2) What could prevent some being able to engage in **Life Conference (Advance care planning)** for you and your family member (the older adult)?

-> How do you think we can minimise these obstacles?

**Questions will be given based on COM-B model (capability, opportunity, and motivation) and Ecological Systems Theory (microsystem, mesosystem, exosystem, macrosystem and chronosystem)*

**5. Needs on ACP (COM-B system & Ecological Systems Theory)**

1) What kind of practical support do you/family need, or did you/family get by health and social care practitioners (especially by homecare managers) to be ready to talk about **Life Conference (Advance care planning)**?

2) What kind of practical support do you/family need, or did you/family get from other aspects (e.g. family, community support, healthcare systems, policy or other social care systems) to be ready to talk about **Life Conference (Advance care planning)**?

**Questions will be given based on COM-B model (capability, opportunity, and motivation) and Ecological Systems Theory (microsystem, mesosystem, exosystem, macrosystem and chronosystem)*

**6. Conclusion questions**

1) Is there anything you would like to share?

2) Do you have any questions?

*That concludes our interview.*

*Thank you very much for taking the time to speak with me today.*

*If you have any further questions or need clarification, please do not hesitate to contact me using the details provided in the participant information sheet.*

*Throughout the interview, ACP would be changed depending on the participants’ understanding. (E.g. Life Conference, having conversations about your preferences and wishes on current and future care and treatment and others)

**Topic guide (Homecare managers, home-visit nurses, homecare physicians)**

*Thank you very much for taking the time to speak with me today.*

*The length of the interview would be approximately 60 mins.*

*If you feel unwell, or if you want to stop the interview, please do let me know. You do not have to give a reason.*

*First of all, could you share some personal information?*

**1. Personal information**

1) May I ask your age?

2) May I ask how many years of experience you have as a homecare manager?

4) Could you tell me about your total years of clinical experience and your previous work history?

*During this interview, I’d like to ask you some questions for you. There is no right or wrong answer to any of my questions. So please share your honest opinion with me. Are you ready to start?*

**2. Explore understanding of advance care planning (ACP)**

1) Have you ever heard of the term **Life conference**, **advance care planning**, advance directives and living will?

* **Life Conference** is the name created by the Ministry of Health, Labour and Welfare as an interpretation of ACP, aimed at making it more understandable for the general public.

2) What kind of information and knowledge do you have related to ACP?

3) Share the definition of ACP in this study, and show the card of the definition below.

***What is Life Conference (Advance care planning)?**

“An individual **thinking** and **discussing** with his or her **family** and other people close to them with the support as necessary of **healthcare providers** who have established a **trusting relationship** with the individual **concerning preparations for the future**: his or her **current state of health and future way of life** and **medical treatment and care** that the individual wishes to receive in the future (Miyashita et al, 2022)”

*Ministry of health, Labour and Welfare named ACP as Life Conference to make it more familiar to the public.

**3. Perspectives and experience with ACP**

1) What do you think about “Life Conference (Advance care planning)”? Why?

2) Have you ever had conversations related to “Life Conference (Advance care planning)” with your clients?

-> If so, what did you discuss? with whom? When? Why?

-> If not, Why?

**4. Facilitators and barriers on ACP (COM-B system & Ecological Systems Theory)**

1) What would enable to be ready to talk about **Life Conference (Advance care planning)** for you/your clients/family carers?

2) What could prevent some being able to engage in ACP for you/your clients/family carers?

-> How do you think we can minimise these obstacles?

**Questions will be given based on COM-B model (capability, opportunity, and motivation) and Ecological Systems Theory (microsystem, mesosystem, exosystem, macrosystem and chronosystem)*

**5. Needs on ACP (COM-B system & Ecological Systems Theory)**

1) What kind of practical support do you need, or did you get as professionals to be ready to talk about **Life Conference (Advance care planning)** ?

2) What kind of practical support do your clients and family to be ready to talk about **Life Conference (Advance care planning)**?

**Questions will be given based on COM-B model (capability, opportunity, and motivation) and Ecological Systems Theory (microsystem, mesosystem, exosystem, macrosystem and chronosystem)*

**6. Conclusion questions**

1) Is there anything you would like to share?

2) Do you have any questions?

*That concludes our interview.*

*Thank you very much for taking the time to speak with me today.*

*If you have any further questions or need clarification, please do not hesitate to contact me using the details provided in the participant information sheet.*

＊Throughout the interview, ACP would be changed depending on the participants’ understanding. (E.g. Life Conference, having conversations about your preferences and wishes on current and future care and treatment and others)

**Appendix 3: Generated themes, sub-themes, and codes**

| **Themes** | **Sub-themes** | **Codes** |
| --- | --- | --- |
| **(1) ‘*Leaving decision-making to the family’* & Bridging family dynamics** | Trust-building relationships as foundations for ACP **(Opportunity)** | Discussions with trusted individuals, regardless of professional role (OA & FC) |
|  |  | ACP following the establishment of trusting relationships (CM & NS) |
|  |  | The gradual process of building trusting relationships (CM & NS) |
|  |  | Professionals' careful attention to building trusting relationships (CM & NS) |
|  |  | Possibility of trust not being established despite time investment (CM) |
|  | The influence of relational authority and communication styles **(Opportunity)** | Older adults with thoughts and preferences for the future (OA & CM) |
|  |  | Variation in conversation content based on relationships (CM & Phys) |
|  |  | Older adults who rarely express their true feelings (CM & NS) |
|  |  | Expectation of implicit understanding (OA & FC) |
|  | Family involvement based on family relationship assessment and power dynamics **(Opportunity)** | Facilitating ACP through smooth family communication (OA, FC, CM, & Phys) |
|  |  | Challenges of ACP in the context of complex family relationships (Phys & CM) |
|  |  | The burden of surrogate decision-making on family members (FC) |
|  |  | Differences of opinion and unspoken topics within families (OA, FC, CM, NS, & Phys) |
|  |  | The potential for family influence to outweigh older adults' autonomy (CM) |
|  |  | The tendency to engage with family members before consulting older adults (FC, CM, & NS) |
|  |  | Assessing family relationships and support capacity (CM) |
|  |  | Negotiating discussions while balancing power dynamics between older adults and family members (NS & CM) |
|  |  | Professionals as advocates for older adults in meetings with family members (CM) |
|  | Securing psychological safety of older adults **(Opportunity)** | Normalising ACP (CM & Phys |
|  |  | Discussions using simple, accessible language (CM & OA) |
|  |  | Everyday conversations about ACP without a serious tone (OA, FC, CM, NS, & Phys) |
|  |  | Using relatable examples for older adults (CM, NS, & Phys) |
|  |  | Ensuring ongoing discussions and continuous support (OA, FC, CM, NS, & Phys) |
|  |  | Assurance that changing one’s wishes is acceptable (NS & Phys) |
|  |  | Assessing the timing and content of discussions (Phys & CM) |
|  | Acceptance of physical decline and delegation of decision-making **(Motivation)** | Anxiety about how long the current lifestyle can be maintained (OA, FC, & CM) |
|  |  | Acceptance of physical decline (OA & FC) |
|  |  | Difficulty envisioning the future (OA, FC, & Phys) |
|  |  | The importance of maintaining daily life over future planning (OA, FC, &CM) |
|  |  | Delegating decision-making to trusted family and accepting their choices (OA & NS) |

| **(Continued) Appendix 3** |  | |  | |  |
| --- | --- | --- | --- | --- | --- |
| **Themes** | **Sub-themes** | | **Codes** | |  |
| **(Continued)**  **(1) ‘*Leaving decision-making to the family’* & Bridging family dynamics** | The influence of older adults’ past experiences and ways of life **(Motivation)** | | Impact of experiences with caregiving, end-of-life care, and physical decline (OA, FC, & CM) | |  |
|  |  |  | Variability in ACP acceptance based on life experiences and personality (OA, FC, & CM) | |  |
|  | Diverse forms of future preparation and awareness of ACP **(Motivation)** | | Diverse perceptions of ACP (OA, CM, NS, & Phys) | |  |
|  |  |  | Diverse forms of future preparation (OA, FC, CM, NS, & Phys) | |  |
| **(2) ‘*ACP is not part of our role’*: Diverging role expectations among professionals, and hesitation to engage in ACP** | The impact of older adults' limited physical and cognitive abilities on ACP **(Capability)** | | Challenges in discussions due to hearing impairment in older adults (OA & FC) | |  |
|  |  |  | Challenges of literacy skills in older adults (OA & CM) | |  |
|  |  |  | Challenges in providing information due to older adults' limited comprehension (FC, CM, NS, & Phys) | |  |
|  | Lack of knowledge about ACP and variations in understanding **(Capability)** | | Knowledge gaps regarding ACP (OA, FC, CM, & NS) | |  |
|  |  |  | Variability in understanding of ACP (CM, NS, FC, Phys, & OA) | |  |
|  | Divergence in the perception of role expectations for professionals **(Motivation)** | | The need for professionals to initiate ACP (OA & FC) | |  |
|  |  |  | Concerns about the psychological burden on older adults (FC, CM, &NS) | |  |
|  |  |  | ACP initiated only when prompted by older adults or their family members (CM & NS) | |  |
|  |  |  | Recognition of homecare managers’ familiarity with older adults and their families (FC, CM, & NS) | |  |
|  |  |  | Perception that ACP is not within the homecare manager’s role (CM) | |  |
|  | Reluctance to engage in ACP among older adults with frailty **(Motivation)** | | Assessing the timing and content of discussions (Phys & CM) | |  |
|  |  |  | Challenges in timing ACP for older adults with frailty (NS, Phys, & CM) | |  |
|  |  |  | Conducting ACP after health deterioration (OA, FC, NS, CM, & Phys) | |  |
|  |  |  | Conducting ACP at end-of-life (CM, NS, & Phys) | |  |
|  |  |  | Tendency to miss opportunities for discussions with older adults with frailty (NS & Phys) | |  |
| **(3) Transitional period: Social norms around death and dying, family structures, and health and social care systems** | | Challenges in collaboration due to hierarchy within multidisciplinary teams **(Mesosystem & Exosystem)** | | Differences of opinion among multidisciplinary teams (CM & Phys) | |
|  |  |  |  | The presence of hierarchy among multidisciplinary teams (CM & NS) | |
|  |  |  |  | Lack of communication with hospitals (CM) | |
|  |  |  |  | Challenges in communicating with physicians (CM, NS, & Phys) | |
|  |  |  |  | Nurses as facilitators of multidisciplinary collaboration (CM & NS) | |
|  |  |  |  | Fostering multidisciplinary relationships through training (CM & NS) | |
|  |  |  |  | Equal relationships among multidisciplinary teams (CM, NS, & Phys) | |
|  |  |  |  | Information sharing through online platforms (NS & Phys) | |

| **(Continued) Appendix 3** |  |  |  |
| --- | --- | --- | --- |
| **Themes** | **Sub-themes** | **Codes** |  |
| **(Continued)**  **(3) Transitional period: Social norms around death and dying, family structures, and health and social care systems** | Limited health and social resources and constraints in information access **(Exosystem)** | Shortage of home doctors and limited end-of-life care experience among homecare managers (CM, NS, & Phys) |  |
|  |  | Disparities in service quality across regions and offices (CM) |  |
|  |  | Lack of information on social resources (OA & FC) |  |
|  |  | Shortage of home doctors and limited end-of-life care experience among homecare managers (CM) |  |
|  |  | Limitation of Long-term Care Insurance system (FC & CM) |  |
|  |  | Diverse literacy levels and economic situations among older adults (CM) |  |
|  | Absence of structured awareness initiatives for ACP **(Exosystem & Macrosystem)** | Promoting awareness of ACP among the public (OA, FC, CM, NS, & Phys) |  |
|  |  | ACP training for professionals across roles and organisations (CM & NS) |  |
|  |  | ACP guidelines and tools (OA, FC, CM, NS, & Phys) |  |
|  |  | Opportunities to understand ACP through case examples (OA, FC, & CM) |  |
|  | Diversified perspectives and attitudes toward death and dying **(Macrosystem)** | Gender roles in decision-making (OA) |  |
|  |  | Diverse attitudes towards discussions involving the context of death (OA, FC, CM, & NS) |  |
|  |  | Conservative tendencies and resistance to new ideas or outsiders (FC & CM) |  |
|  | Diversified of family structures and family role expectations **(Macrosystem)** | Diverse family structures and care roles (CM) |  |
|  |  | Challenges in securing time for discussions with family carers (CM, NS, & Phys) |  |
|  |  | Diverse role expectations in family caregiving (OA, FC, & CM) |  |
| *OA: older adults; FC: family caregivers; CM; homecare managers; NS: home-visit nurses; Phys: homecare physicians | | | |
