# Supplementary material for: Exploring readiness for advance care planning in Japan: A qualitative interview study with older adults with frailty, family caregivers, and health and social care practitioners in the homecare setting
Source: Palliat Care Soc Pract. 2025 Nov 28;19:26323524251395654. doi: 10.1177/26323524251395654 (PMC12665015; doi:10.1177/26323524251395654)
Supplement: sj-pdf-1-pcr-10.1177_26323524251395654 – Supplemental material for Exploring readiness for advance care planning in Japan: A qualitative interview study with older adults with frailty, family caregivers, and health and social care practitioners in the homecare setting [file sj-pdf-1-pcr-10.1177_26323524251395654.pdf]

Reporting guideline: Reflexive Thematic Analysis Reporting Guidelines (RTARG) checklist

Guideline Items	How it was addressed
**1. The Introduction**	
**1.1 Background and rationale**	
1.1.1. Provide a robust context and rationale for the proposed research in the Introduction.	A robust rationale was provided through discussion of relevant research and theories, along with a description of the context of the research site in background section.
1.1.2. Clearly articulate a research question – one that is methodologically coherent.	The research aims and objectives aligned with a qualitative methodology, which allows for in-depth exploration of participants' experiences and perspectives on readiness for ACP.
**1.2 "Owning your perspectives"**	
1.2.1. Include information on guiding theoretical assumptions and other (e.g., explanatory) theory informing the use of TA.	This study is guided by a pragmatic approach, which supports the use of reflective thematic analysis to flexibly explore real-world experiences and practical implications. In line with pragmatism, the research question is prioritised over specific methods or paradigms [1], allowing for a focus on meaningful, actionable insights into readiness for ACP. The study is also grounded in the COM-B system[2], which provides a framework for understanding individual behaviour change mechanisms, and Ecological Systems Theory[3], which situates readiness within broader social and environmental contexts. These frameworks informed every stage of the research process, from shaping the research aims to guiding data collection and analysis. By integrating these theories, the study offers a comprehensive understanding of the factors influencing readiness for ACP, aligned with its pragmatic goal of generating actionable and contextually relevant insights.
1.2.2. Report in a way that is consistent with stated theoretical assumptions throughout.	The report maintains theoretical coherence by consistently applying language and concepts aligned with reflexive thematic analysis. Research subjectivity was acknowledged through the use of field notes, reflexive journaling, and self-positioning statements, which enabled critical engagement with the data and enhanced interpretative transparency by recognising the researcher's influence on theme development and data interpretation.
1.2.3. Evidence methodological coherence/integrity in both the research and the report.	Methodological coherence was achieved by aligning the theoretical assumptions, research aim, methods of data generation, and the use of reflexive thematic analysis within a pragmatic framework.

(Continued) Appendix 1

Guideline Items	How it was addressed
1.2.4. Show evidence of reflexive practice.	In our study, reflexive practice was evidenced through a critical examination of the researchers' professional and personal positioning in relation to the research topic and participant group, as outlined in this table. For instance, the primary researcher (MF), a Japanese national with a nursing background, drew on personal experiences in oncology nursing at an acute tertiary care hospital and five years of research in ACP, including questionnaire surveys and interview studies. These experiences informed the research focus and shaped the interpretative lens throughout research process. Reflexivity was further enhanced through collaboration with researchers from diverse backgrounds: IN, a Certified Nurse Specialist in Home Care Nursing with over 10 years of experience as a home visiting nurse at the research site; YS, a psychology professor specialising in bereavement care; and CJE and JK, British professors specialising in palliative care and qualitative studies.<br>Field notes and reflexive journaling were employed throughout the research process to identify and critically reflect on how the researchers' perspectives shaped the study. These practices enabled us to examine potential influences of our positioning on data interpretation and analysis. Reflexive practices are explicitly integrated into the research narrative and further detailed in this table.
1.2.5. Write in a methodologically coherent style.	A third-person writing style was adopted in line with common practices in the journal to ensure clarity and reader-friendliness. However, the manuscript was carefully crafted to incorporate language and concepts relevant to reflexive thematic analysis, ensuring alignment with its principles and avoiding a positivist tone.
**2. The Methodology**	
**2.1. Participants/data items**	
2.1.1. Describe selection of participants/data items.	It was demonstrated this in **2.3 Setting and Participants**, and **2.4 Selection of participants.**
2.1.2. Describe number of participants/data items; provide a rationale or explanation around dataset or participant group size/composition.	It was detailed it in **2.4 Selection of participants**. Individual interviews with professionals were conducted, as MF anticipated that some might not feel safe sharing their honest views due to the hierarchy among professionals, based on MF's clinical experience.
2.1.3. Discuss characteristics of participants/data items.	It was provided participants' demographic background in **Table 3: Characteristics of Participants.** However, Anonymity was ensured by presenting aggregated information and omitting specific identifiers where appropriate.
2.1.4. Detail ethical approval and ethical code/principles followed, participant informed consent, etc.	It was described it in **2.8 Ethical approval.**

(Continued) Appendix 1

Guideline Items	How it was addressed
**3. Dataset generation**	
3.1. Provide some rationale for method(s) for data generation/data item sources chosen.	Multiple stakeholders were included to gain a complex, in-depth understanding of readiness for ACP. This approach aligns with the research aim and objectives, which focus on understanding the experiences and perspectives related to ACP readiness in frail older adults, a process that is inherently dynamic. The COM-B system[2] and the Ecological Systems Theory[3] were used to guide the exploration, providing theoretical frameworks to understand the interplay of individual capabilities, opportunities, motivations, and broader contextual factors.
3.2. Describe development and/or characteristics of data generation tool(s).	We used topic guide and explained how it was developed in **2.6 Data collection.**
3.3. Include details such as modality and/or setting of data generation, time frame, and other pertinent procedural information.	Please refer to **2.6 Data collection**.
3.4. Describe who conducted any interactive data generation (which author or research role), and how.	All interviews were conducted by the researcher (MF), a Japanese female with clinical experience as a nurse and skills in qualitative interviewing. MF's background provided insight into the clinical context, facilitating rapport-building and an empathetic understanding of participants' perspectives. As MF had no prior relationship with the participants, she took time to carefully explain the research purpose and used informal conversations to build rapport. The interviews were conducted in Japanese, audio-recorded, and transcribed in Japanese. Throughout the interviews, MF maintained field notes and engaged in reflexive journaling, capturing both verbal and non-verbal communication with stakeholders and participants from recruitment to completion. MF transcribed the interview data in Japanese and read the transcripts multiple times to gain a thorough understanding of participants' experiences and perceived readiness for ACP. Additionally, she reviewed field notes and reflexive journals, adding further reflections during the analysis process.
3.5 Describe the size/scope of dataset and dataset items.	It is reported in **3.1 participants.** The mean interview duration was 72 minutes (range 49 to 132 minutes).
3.6 Describe, and if relevant explain, any preparation of data for analysis.	Audio data from the interviews was transcribed verbatim in Japanese, maintaining the original language to preserve meaning and context. Minor typographical errors were corrected for readability, ensuring clarity without altering participants' intended meaning. Pseudonyms were assigned to each participant, and these were used consistently throughout the analysis and reporting to maintain confidentiality while allowing for clear participant referencing.

(Continued) Appendix 1

Guideline Items	How it was addressed
**4. Data analysis**	
4.1. Provide some rationale for use of RTA, and, where relevant, for combining RTA with other approaches and procedures.	Reflexive Thematic Analysis is well-suited for capturing the depth and complexity of subjective experiences and perspectives on readiness for ACP. Its flexibility enables researchers to engage deeply with participants' narratives, allowing for an understanding of the nuanced and dynamic factors influencing readiness for ACP.
4.2. Describe specific orientation to RTA.	It was detailed in **2.7 Data analysis.**
4.3. Discuss how the researcher(s) engaged with the analytic process.	It was detailed in **2.7 Data analysis.**
4.4. Where more than one person is involved, describe who analysed the data (author or research role).	Differences in data interpretation or coding were resolved through reflexive discussions among the researchers, ensuring that the themes reflected diverse viewpoints and captured the complexity of readiness for ACP. This collaborative process adhered to the principles of reflexivity, recognising the influence of the researchers' backgrounds and perspectives on the analysis.
4.5. Use language to describe the process and products of RTA that is coherent with the values and assumptions of RTA.	It was carefully reported using language aligned with the values and assumptions of Reflexive Thematic Analysis.
**5. The Analysis**	
**5.1. Reporting the data analysis**	
5.1.1. Provide an overview of themes or thematic structure.	An overview of all themes and sub-themes were provided in **Table 4**, along with the codes by respective theme in **Appendix 3**.
5.1.2. Ensure theme conceptualisation is appropriate to RTA, and any divergences are justified and explained.	It was detailed in **2.7 Data analysis.** Themes were carefully generated and named to ensure they encapsulate shared meaning, organised around a central organising concept, as recommended in the guidelines. Each theme reflects a deeper level of interpretation, going beyond mere topic summaries. In generating the themes, we focused on capturing patterns of meaning that resonate across the dataset, ensuring they are conceptually coherent and distinct from one another through repeated discussions with researchers.
5.1.3. Name themes appropriately.	It was detailed in **2.7 Data analysis.** Themes were named to capture their essence and overarching narrative, moving beyond simple topic identification. Simplistic theme names were avoided, with an emphasis on reflecting the deeper narrative uncovered during analysis and incorporating quotations where appropriate.

(Continued) Appendix 1

Guideline Items	How it was addressed
5.1.4. Report themes in sufficient depth and detail.	Themes were reported with depth and detail in **3.2 Themes**, ensuring they were multifaceted and included both data and analytic narrative, in accordance with the recommendations of reflexive thematic analysis.
5.1.5. Use subtheme judiciously.	Details of how we developed them are provided in **2.7 Data analysis** about how we developed them. Furthermore, detailing of the sub-themes are described in **3.2 Themes.**
5.1.6. Ensure the analytic narrative explains the meaning and significance of the data.	The analytic narrative for each theme was developed to explain its meaning and significance in relation to the topic, research question, and dataset. Each theme was supported by an interpretative narrative that highlighted underlying patterns of meaning. The overall story of the findings, including the positioning of each theme and sub-theme, are presented in **3.2 Themes.**
5.1.7. Provide an appropriate balance of analytic narrative and data extracts – both data extracts and analytic narrative matter.	The analytic narrative and data extracts were balanced to avoid mere narrative summaries, with a focus on demonstrating sufficient and meaningful analytic interpretation.
5.1.8. Demonstrate coherence between analytic narrative and illustrative/evidentiary data extracts.	A clear connection between the analytic narrative and the data extracts was established by selecting examples that strongly supported the claims. Each data extract was explained within the narrative to clarify its importance and relevance. To enhance the credibility and depth of the analysis, alternative interpretations of the data were also considered.
5.1.9. Integrate existing research and theory into the analytic narrative.	Existing research and theoretical frameworks were integrated into the analytic narrative to provide context and enrich the interpretation of themes. The COM-B system[2] and the Ecological Systems Theory[3] were specifically used as theoretical underpinnings to frame the analysis and interpret the findings. These frameworks contextualised the patterns of meaning within the data, offering a deeper understanding of readiness for ACP by considering both individual behaviour change and the influence of external factors. Relevant previous studies were also referenced to enhance the interpretative analytic narrative and reflect contextualised meaning. It was reported in the discussion section in a way that aligns with common practices in the journal to ensure clarity and reader-friendliness.
**6. The Final Section – A General Discussion or "Conclusions"**	
**6.1. Quality, evaluation and conclusions**	
6.1.2. Draw analytic conclusions across themes.	The manuscript ensures that analytic conclusions are drawn across the themes, highlighting the overarching narrative of the analysis in **4. Discussion**.

(Continued) Appendix 1

Guideline Items	How it was addressed
6.1.3. Discuss implications or directions for future research.	It was detailed in **4.4. Implications for clinical practice and research** section.
6.1.4. Use and report quality practices coherent with RTA.	The study employed quality practices aligned with the principles of Reflexive Thematic Analysis to ensure rigour and depth in the analysis. Field notes and reflexive journaling were used throughout the research process to document evolving thoughts and interpretations, enhancing reflexivity rather than seeking consensus or validation. Critical discussions with other researchers provided alternative perspectives, deepening insights and refining the analysis. In reporting, language relevant to Reflexive Thematic Analysis was applied.
6.1.5. Evaluate the research from a Big Q standpoint.	The study employed quality practices aligned with the principles of Reflexive Thematic Analysis to ensure rigour and depth in the analysis. Field notes and reflexive journaling were used throughout the research process to document evolving thoughts and interpretations, enhancing reflexivity rather than seeking consensus or validation. The participant group's characteristics and context, along with the data collection methods, were recognised as integral to shaping the findings, not as limitations. Critical discussions with other researchers provided alternative perspectives, deepening insights and refining the analysis. In reporting, language relevant to Reflexive Thematic Analysis was applied to reflect the situatedness of the study.
6.1.6. Include reflections on research process and practices, including researcher reflexivity.	Researchers' reflections were incorporated into the critical discussion, highlighting how our positionality informed the analytic lens and the construction of themes presented in this table.
